# Supplementary figures and images for: Multiomics analyses reveal dynamic bioenergetic pathways and functional remodeling of the heart during intermittent fasting
Source: eLife. 2023 Sep 28;12:RP89214. doi: 10.7554/eLife.89214 (PMC10538958; doi:10.7554/eLife.89214)

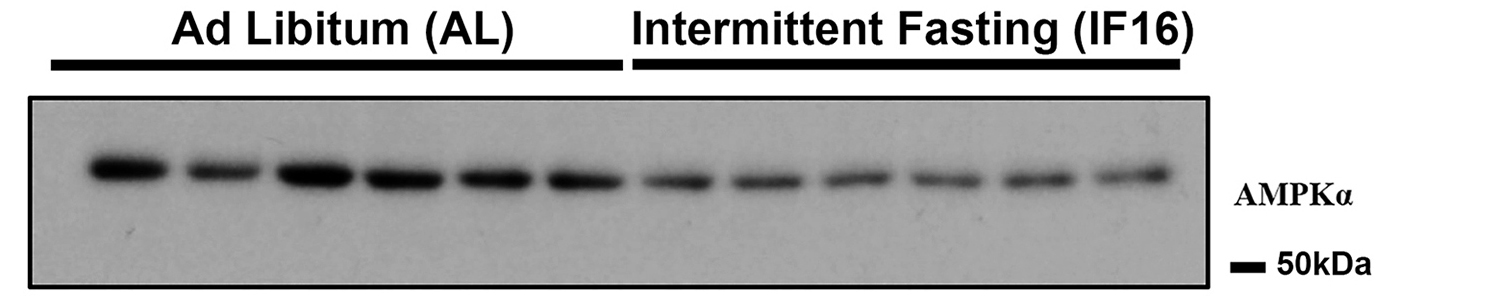

Supplement: Figure 5—source data 1. [file elife-89214-fig5-data1.zip › Figure 5 source data/2 AMPK.jpg]

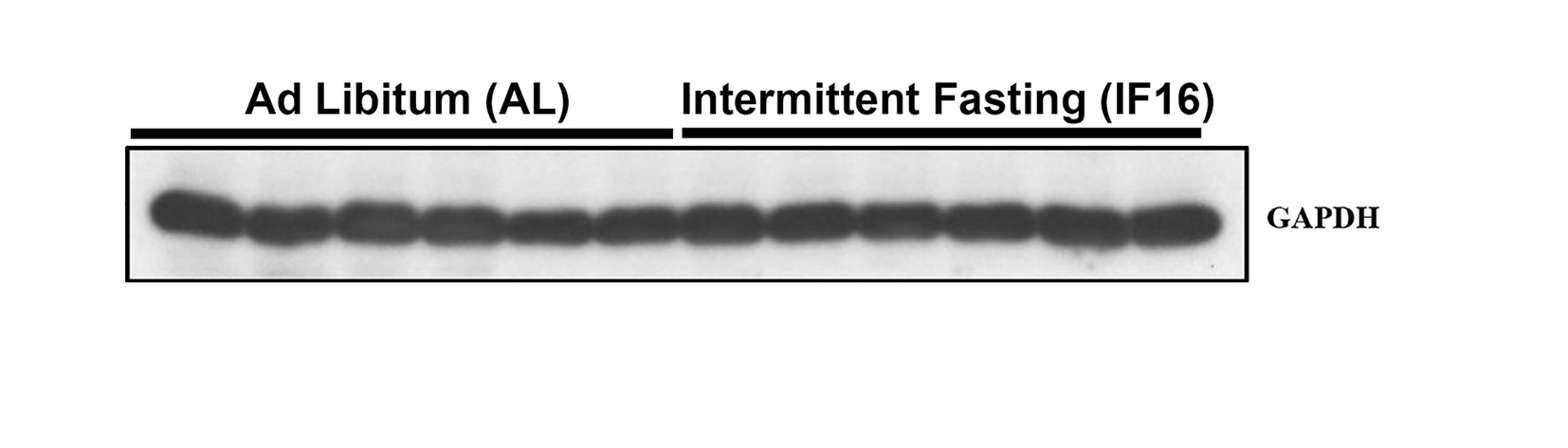

Supplement: Figure 5—source data 1. [file elife-89214-fig5-data1.zip › Figure 5 source data/11 GAPDH.jpg]

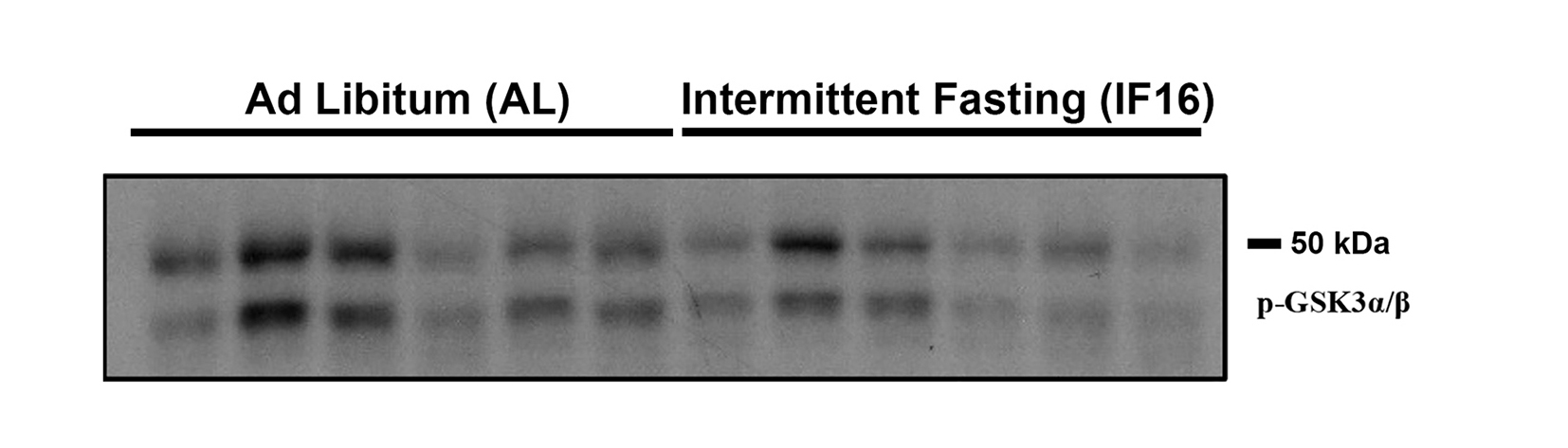

Supplement: Figure 5—source data 1. [file elife-89214-fig5-data1.zip › Figure 5 source data/8 p-GSK3aB.jpg]

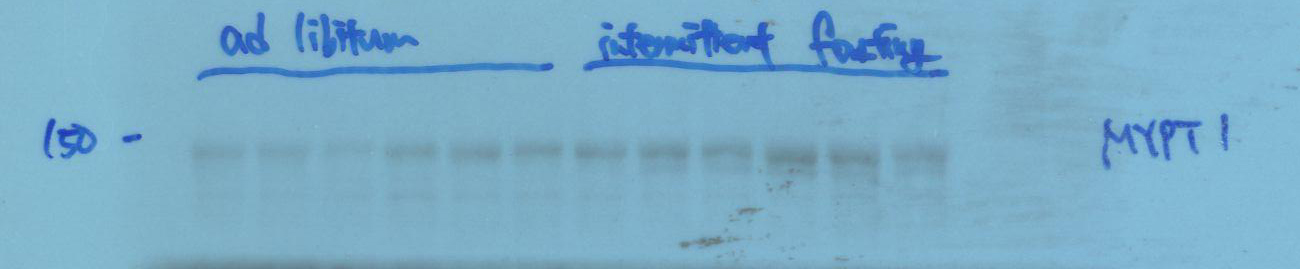

Supplement: Figure 5—source data 1. [file elife-89214-fig5-data1.zip › Figure 5 source data/4 MYPT-1 raw unedited.jpg]

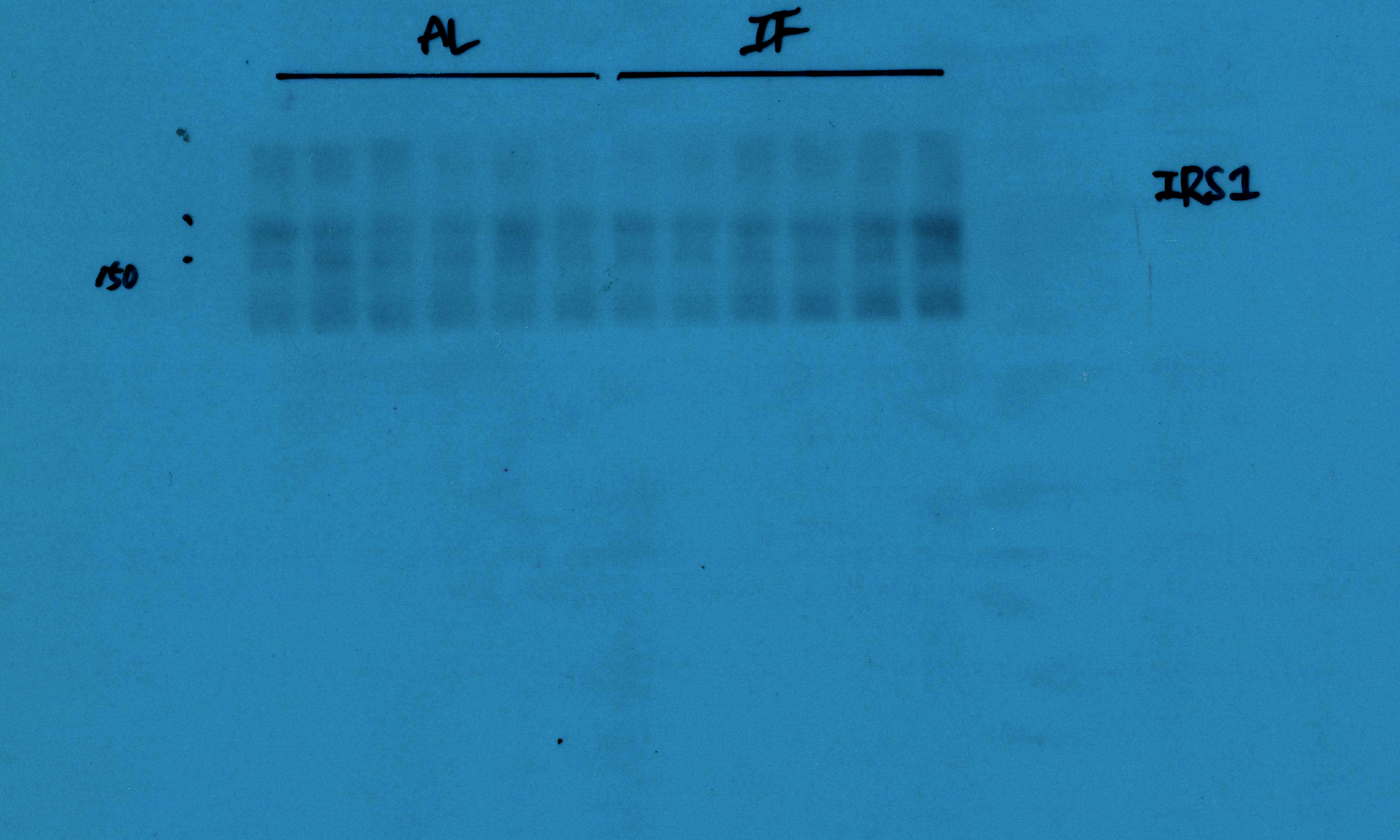

Supplement: Figure 5—source data 1. [file elife-89214-fig5-data1.zip › Figure 5 source data/13 IRS-1 raw unedited.jpg]

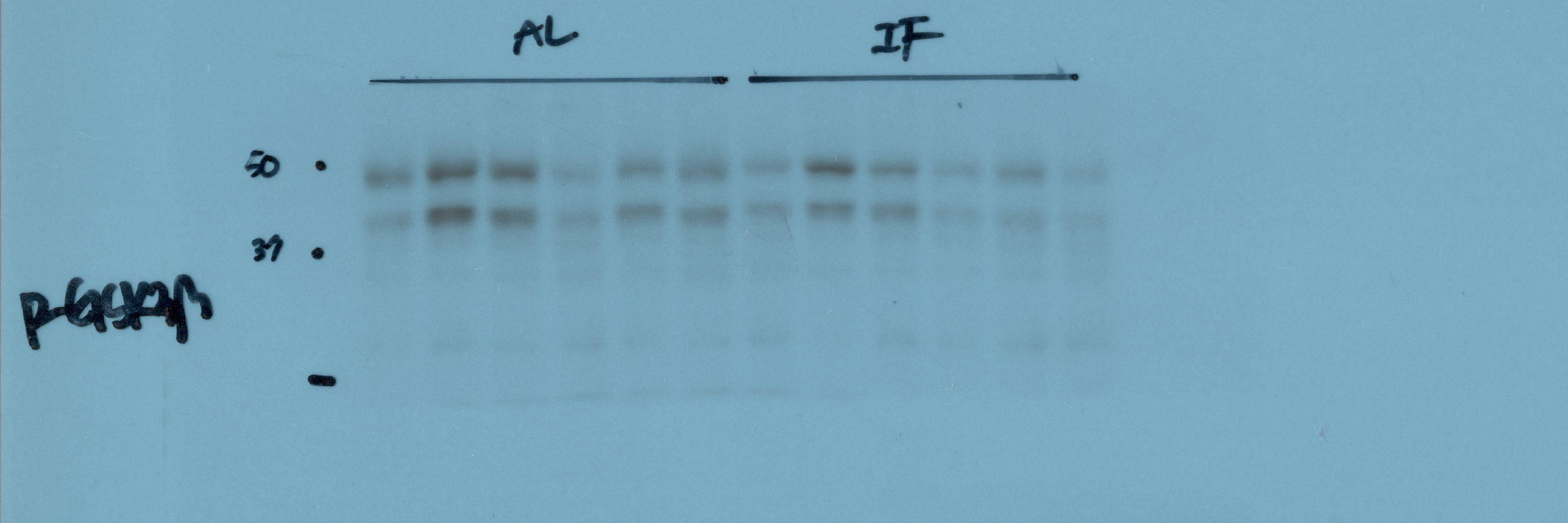

Supplement: Figure 5—source data 1. [file elife-89214-fig5-data1.zip › Figure 5 source data/8 p-GSK3aB raw unedited.jpg]

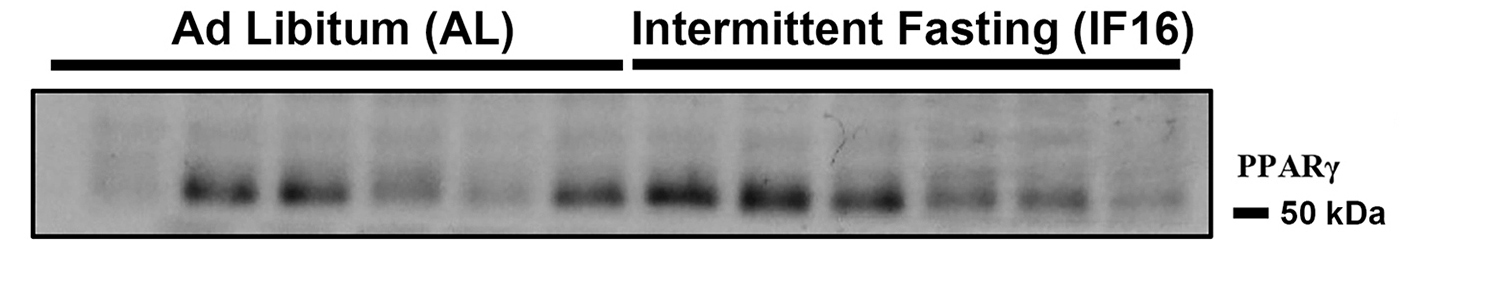

Supplement: Figure 5—source data 1. [file elife-89214-fig5-data1.zip › Figure 5 source data/5 PPARg.jpg]

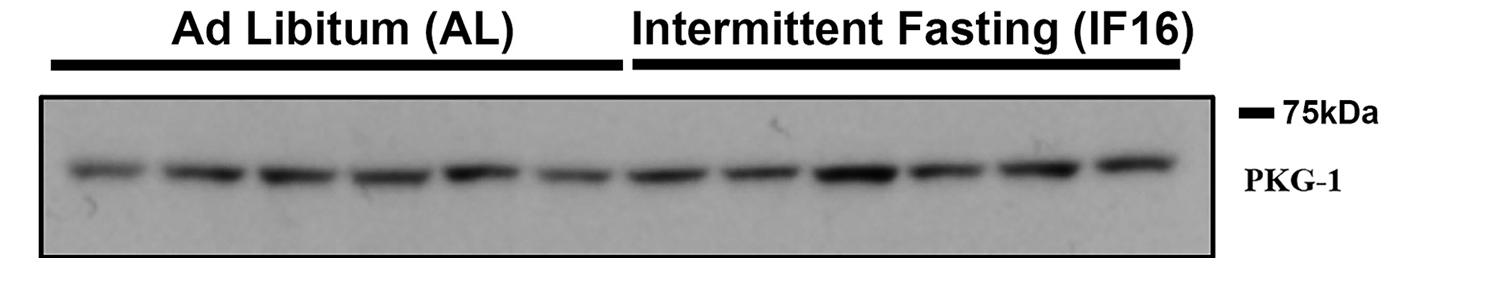

Supplement: Figure 5—source data 1. [file elife-89214-fig5-data1.zip › Figure 5 source data/3 PKG-1.jpg]

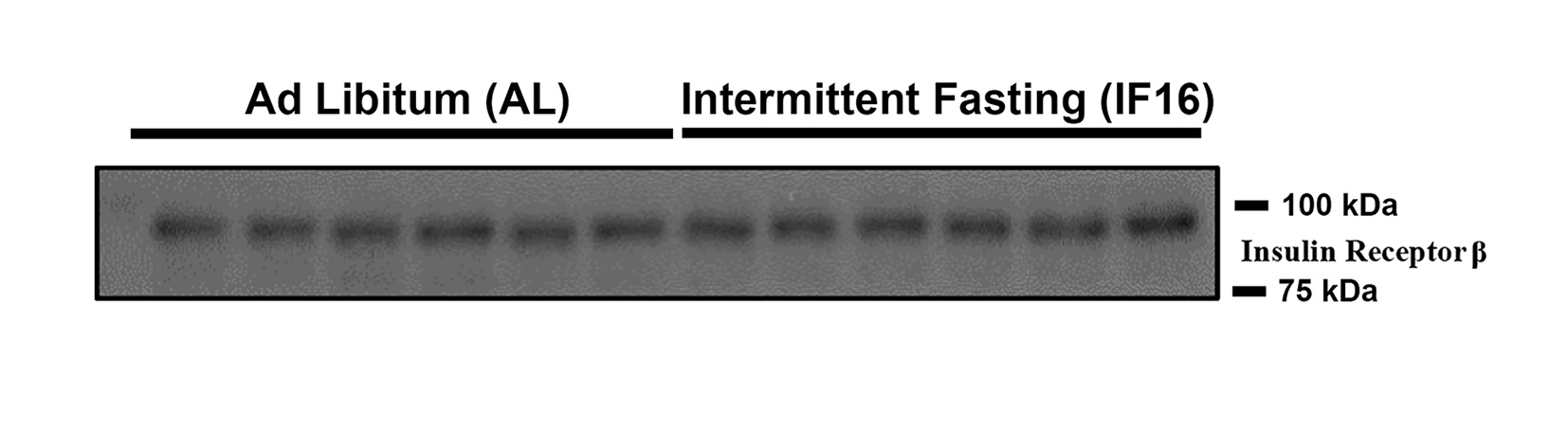

Supplement: Figure 5—source data 1. [file elife-89214-fig5-data1.zip › Figure 5 source data/10 Insulin receptor.jpg]

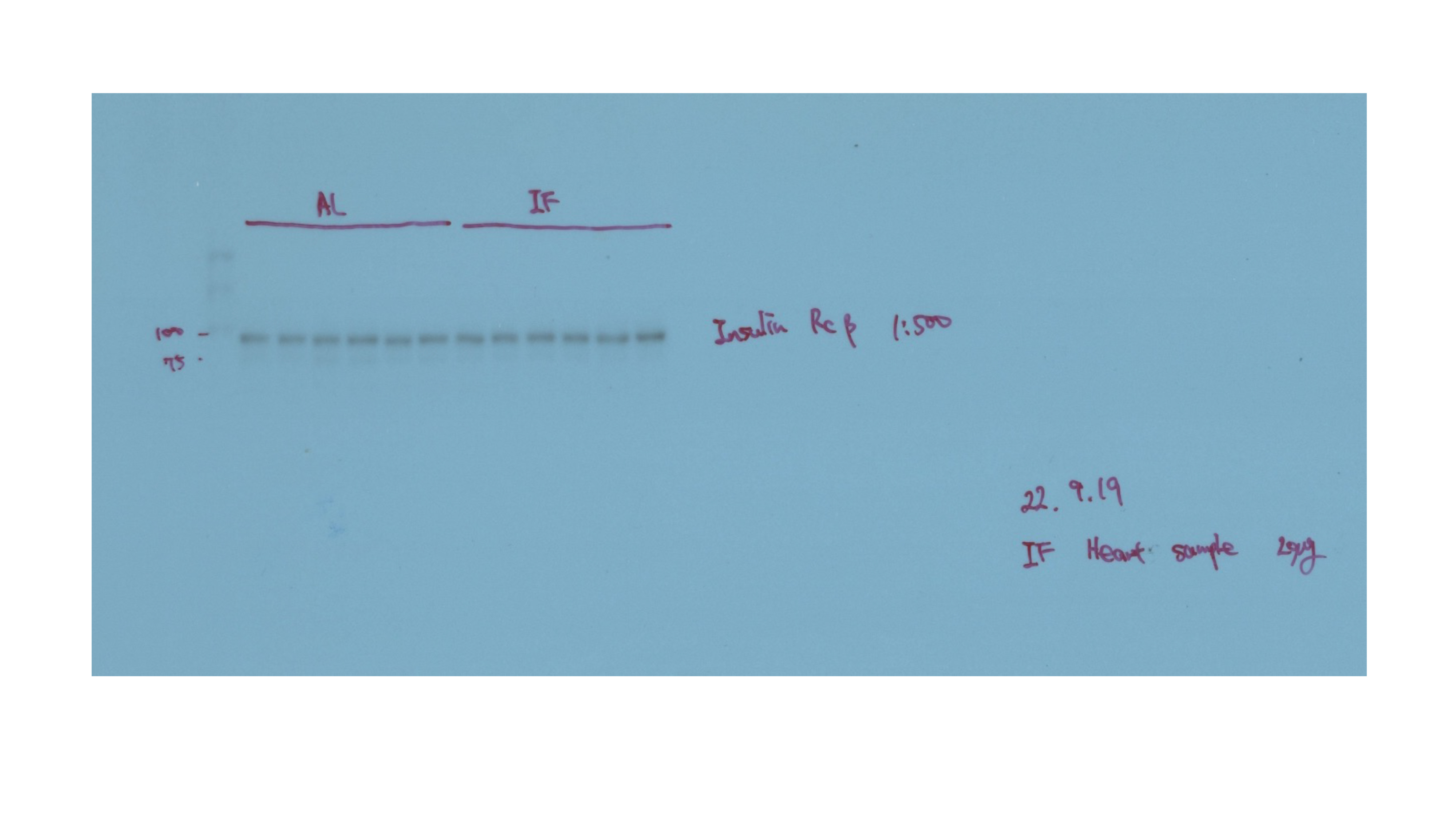

Supplement: Figure 5—source data 1. [file elife-89214-fig5-data1.zip › Figure 5 source data/10 Insulin receptor raw unedited.jpg]

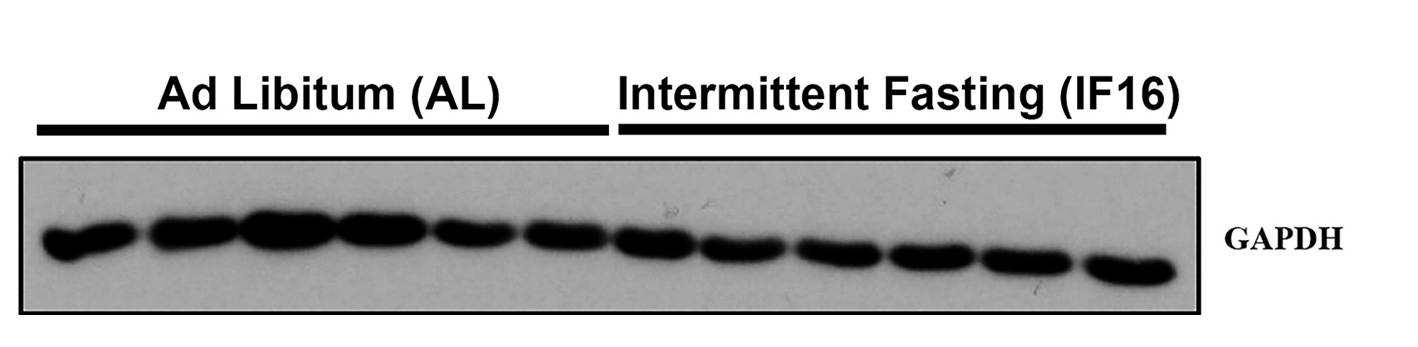

Supplement: Figure 5—source data 1. [file elife-89214-fig5-data1.zip › Figure 5 source data/7 GAPDH.jpg]

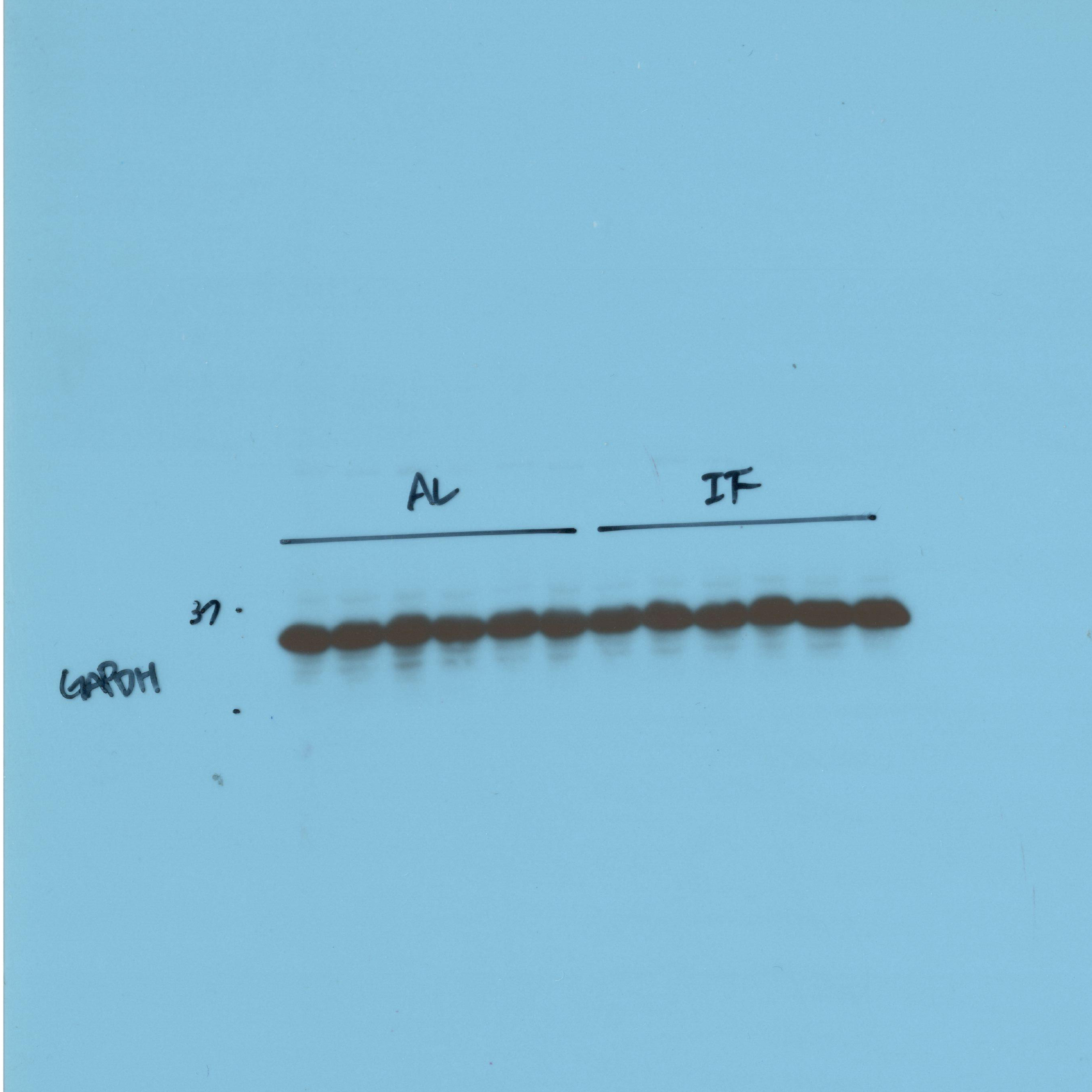

Supplement: Figure 5—source data 1. [file elife-89214-fig5-data1.zip › Figure 5 source data/14 GAPDH raw unedited.jpg]

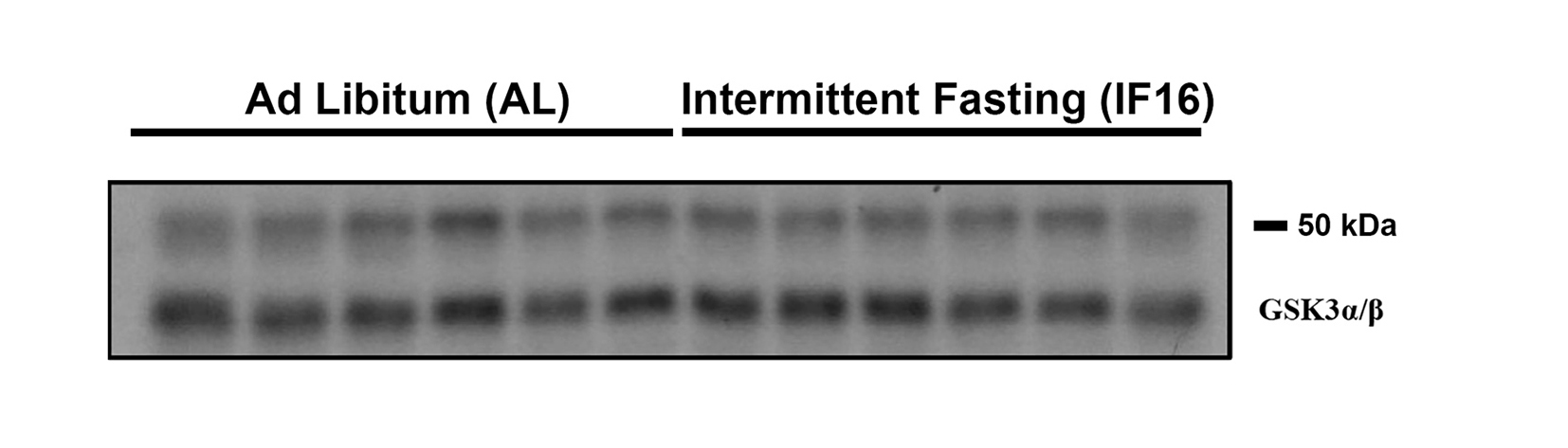

Supplement: Figure 5—source data 1. [file elife-89214-fig5-data1.zip › Figure 5 source data/9 GSK3aB.jpg]

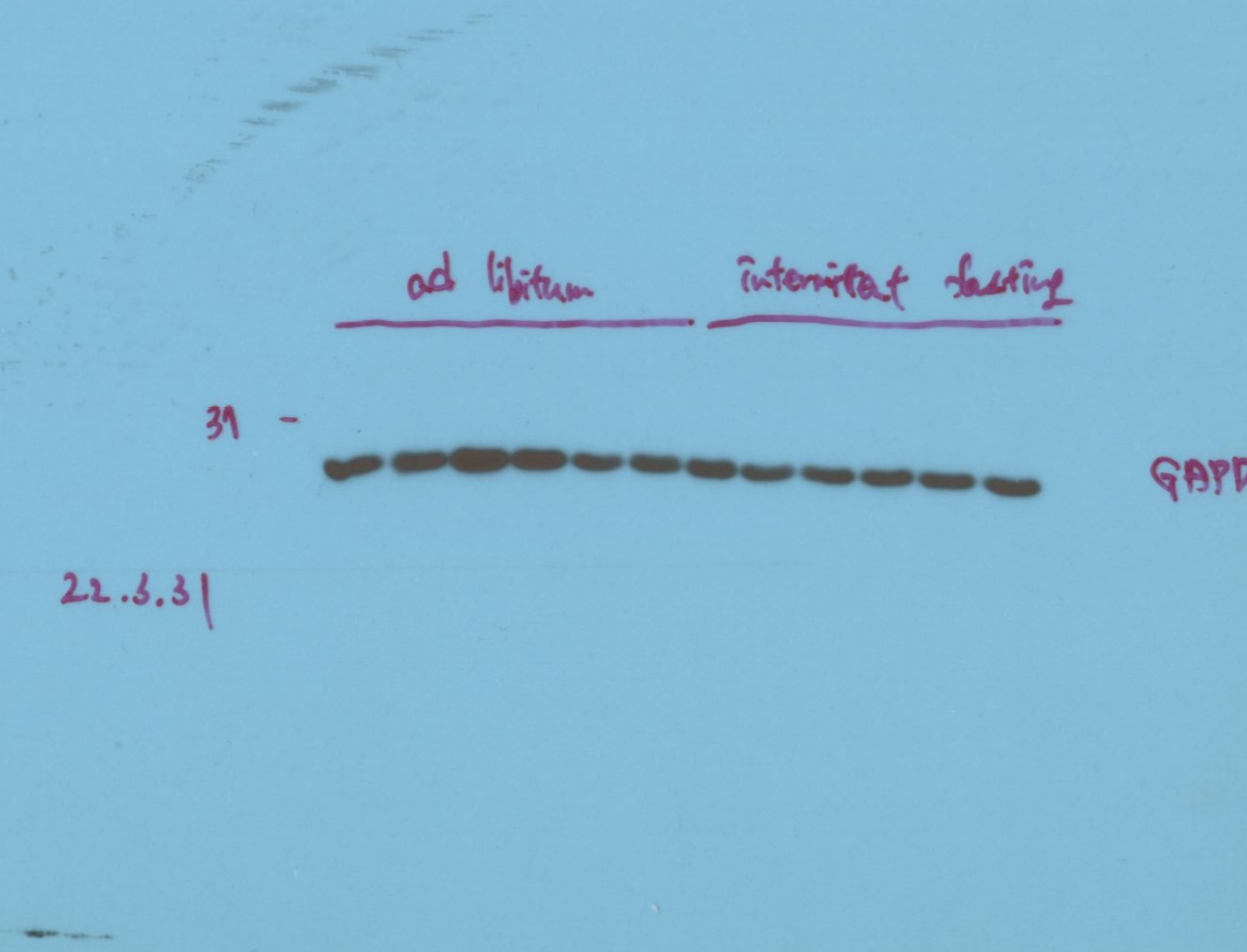

Supplement: Figure 5—source data 1. [file elife-89214-fig5-data1.zip › Figure 5 source data/7 GAPDH raw unedited.jpg]

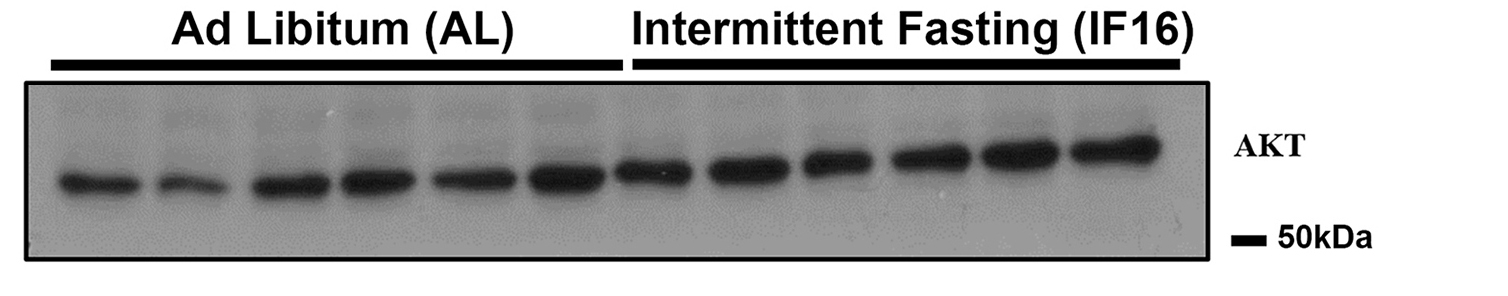

Supplement: Figure 5—source data 1. [file elife-89214-fig5-data1.zip › Figure 5 source data/6 AKT.jpg]

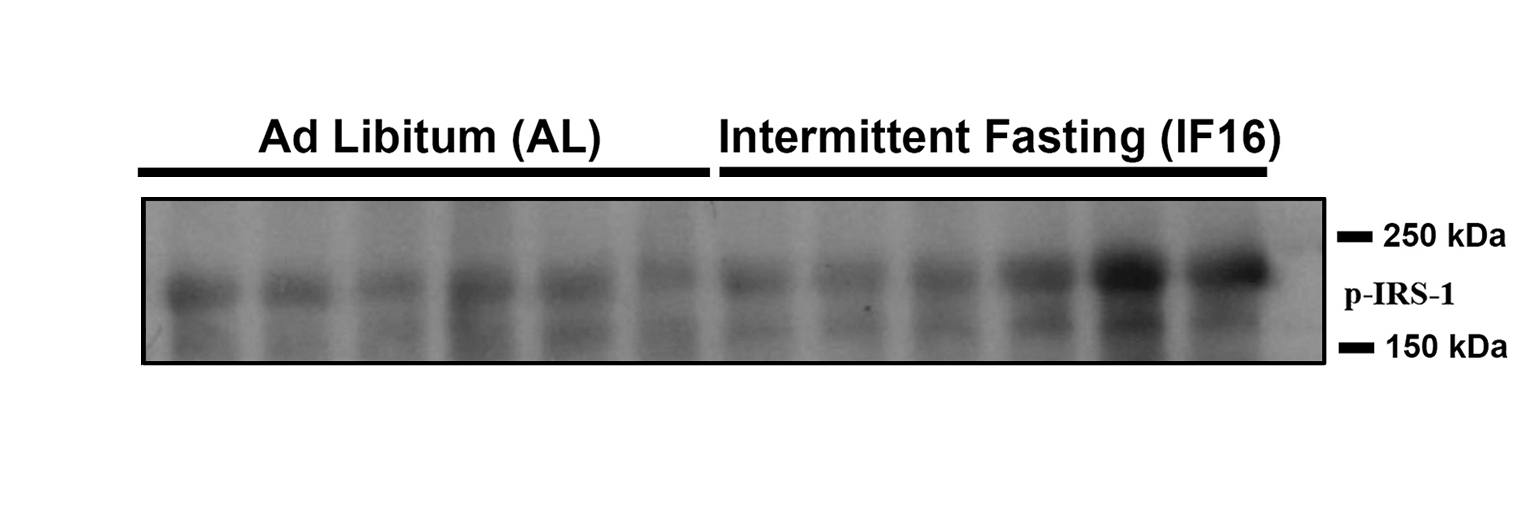

Supplement: Figure 5—source data 1. [file elife-89214-fig5-data1.zip › Figure 5 source data/12 p-IRS1.jpg]

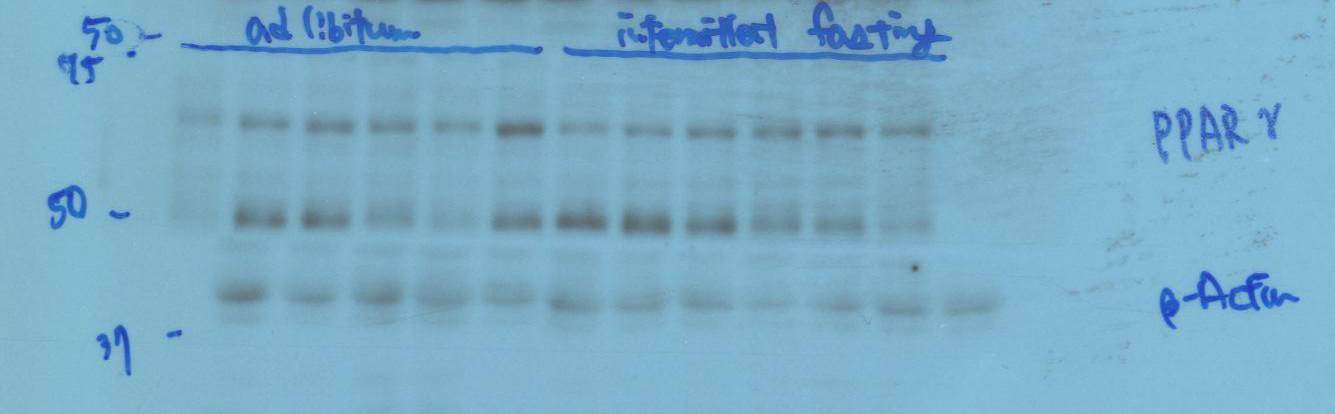

Supplement: Figure 5—source data 1. [file elife-89214-fig5-data1.zip › Figure 5 source data/5 PPARg raw unedited.jpg]

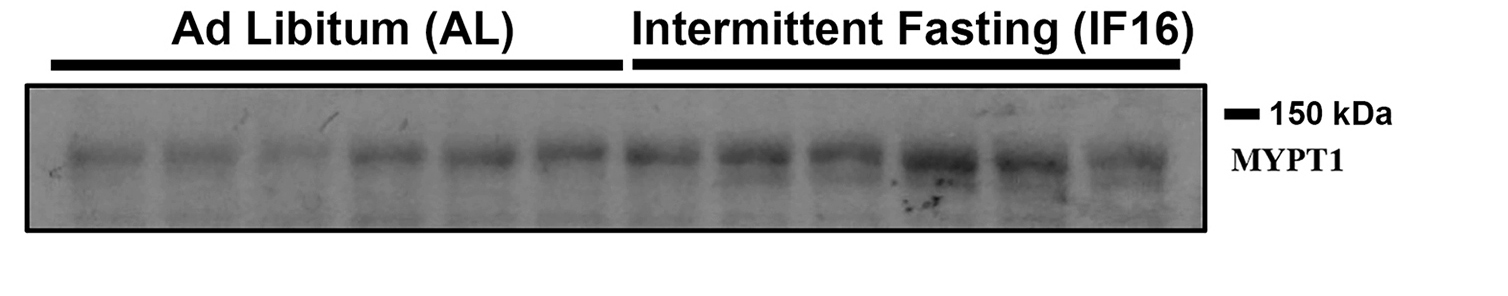

Supplement: Figure 5—source data 1. [file elife-89214-fig5-data1.zip › Figure 5 source data/4 MYPT-1.jpg]

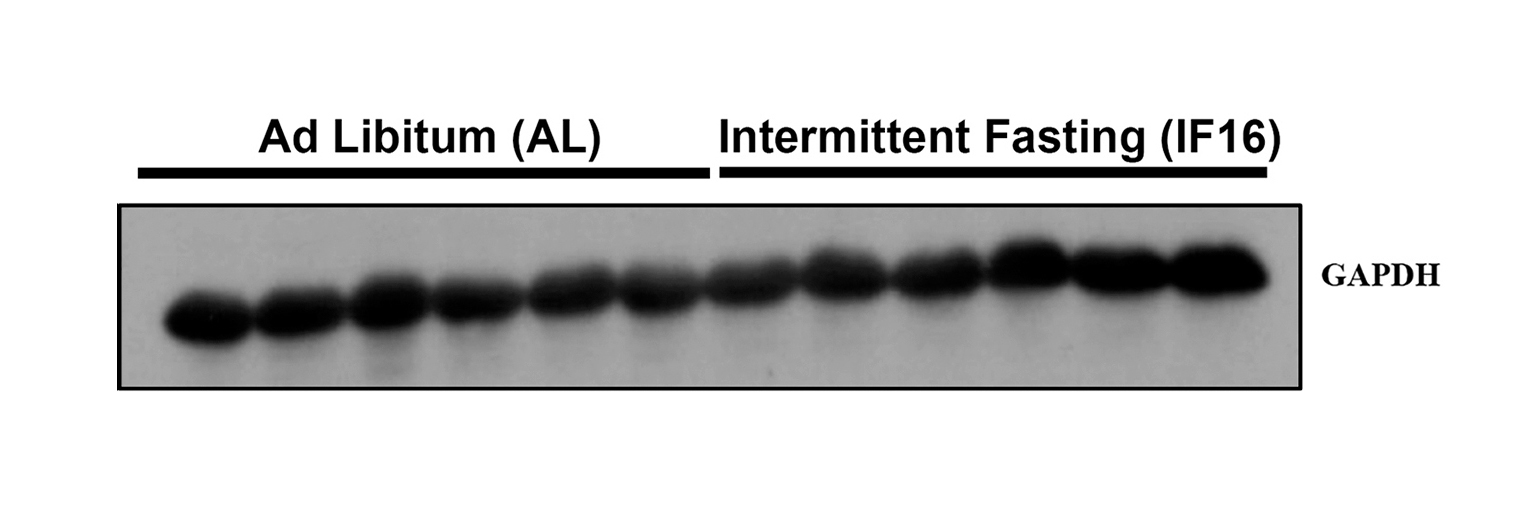

Supplement: Figure 5—source data 1. [file elife-89214-fig5-data1.zip › Figure 5 source data/14 GAPDH.jpg]

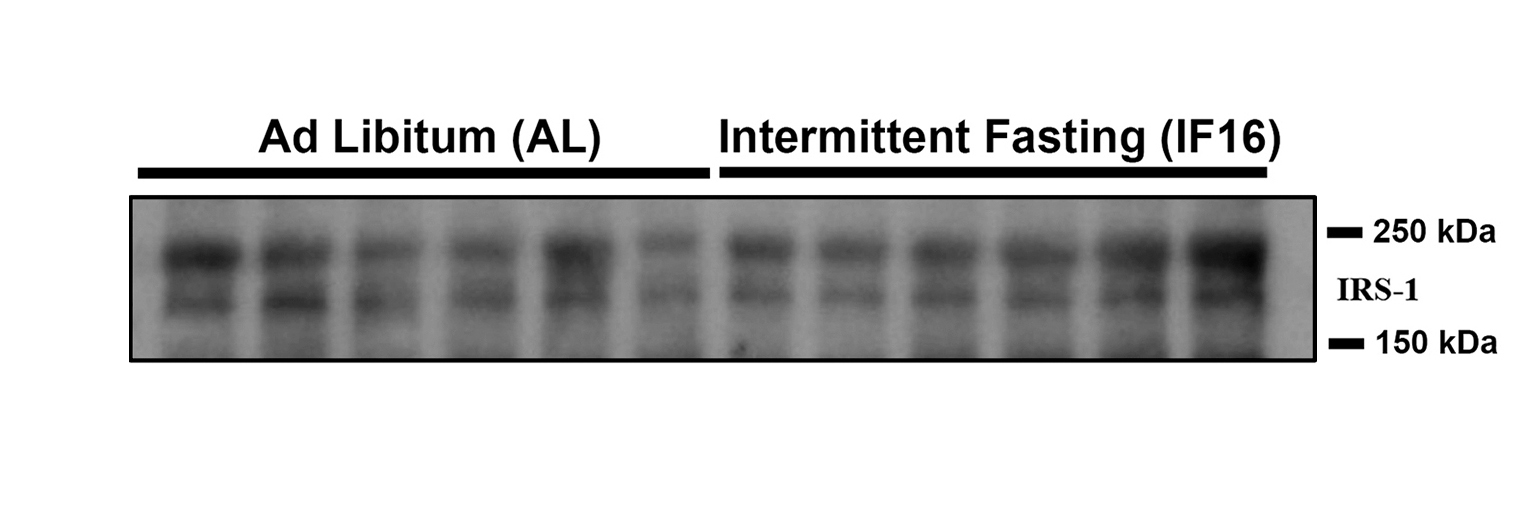

Supplement: Figure 5—source data 1. [file elife-89214-fig5-data1.zip › Figure 5 source data/13 IRS1.jpg]

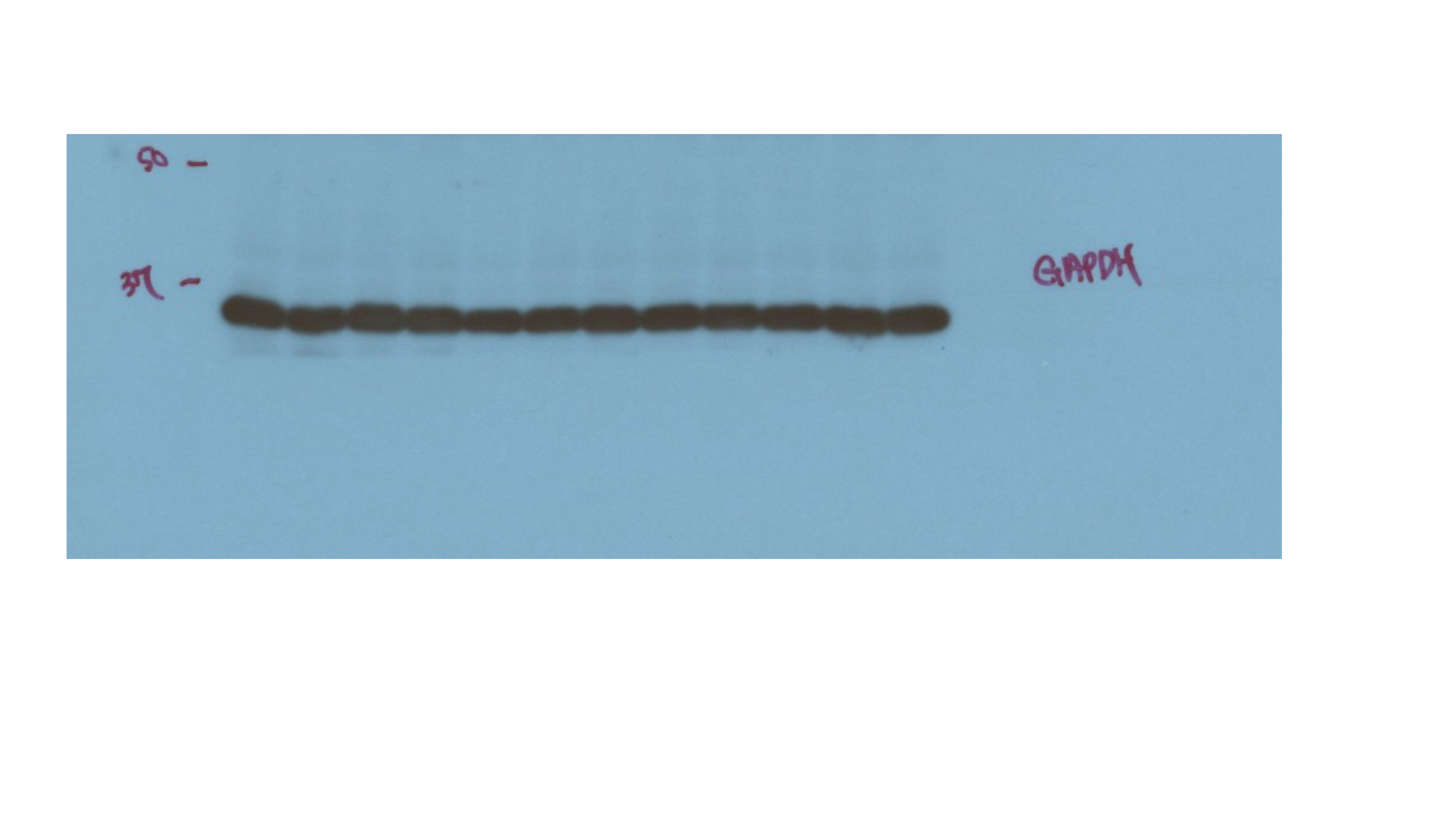

Supplement: Figure 5—source data 1. [file elife-89214-fig5-data1.zip › Figure 5 source data/11 GAPDH raw unedited.jpg]

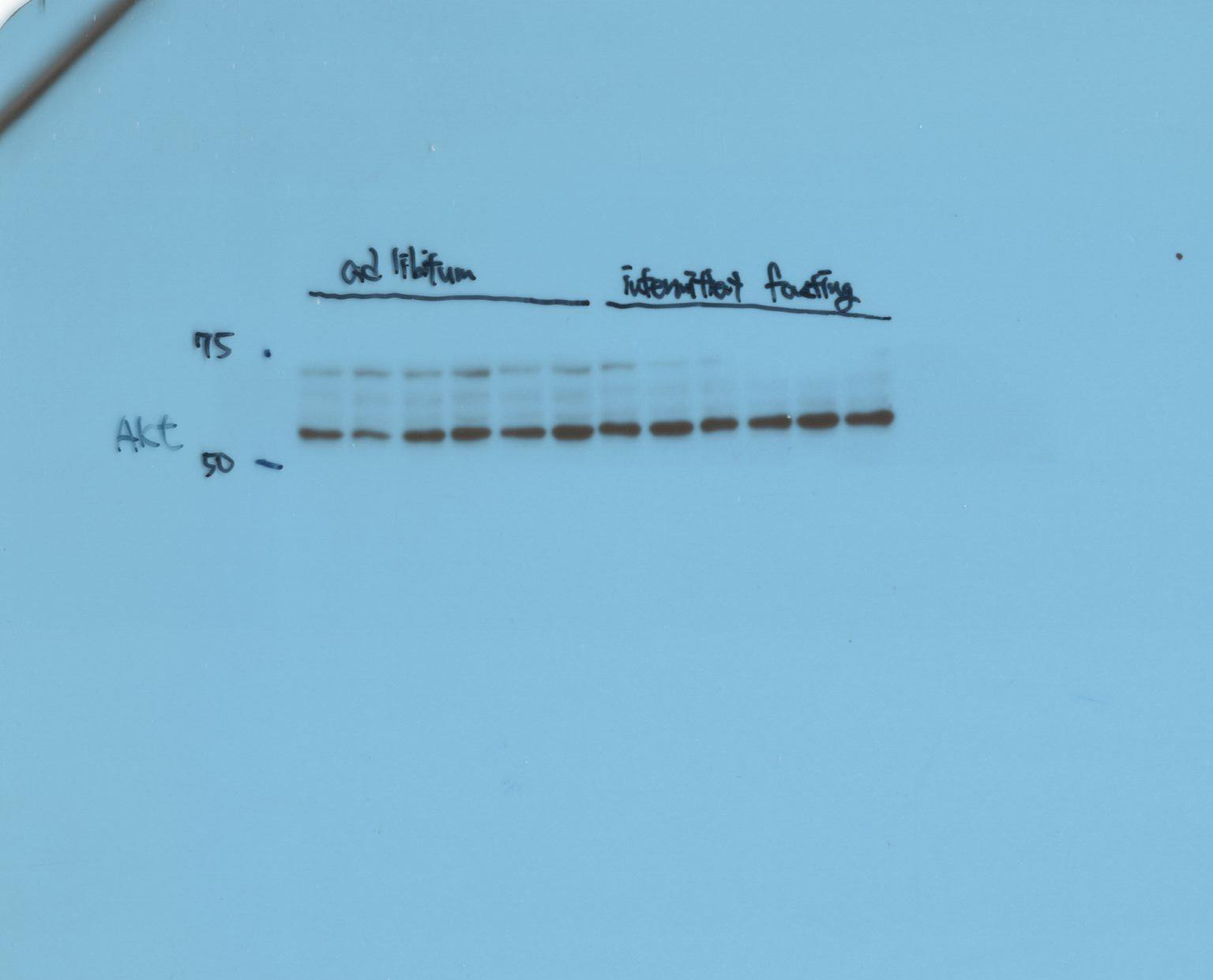

Supplement: Figure 5—source data 1. [file elife-89214-fig5-data1.zip › Figure 5 source data/6 AKT raw unedited.jpg]

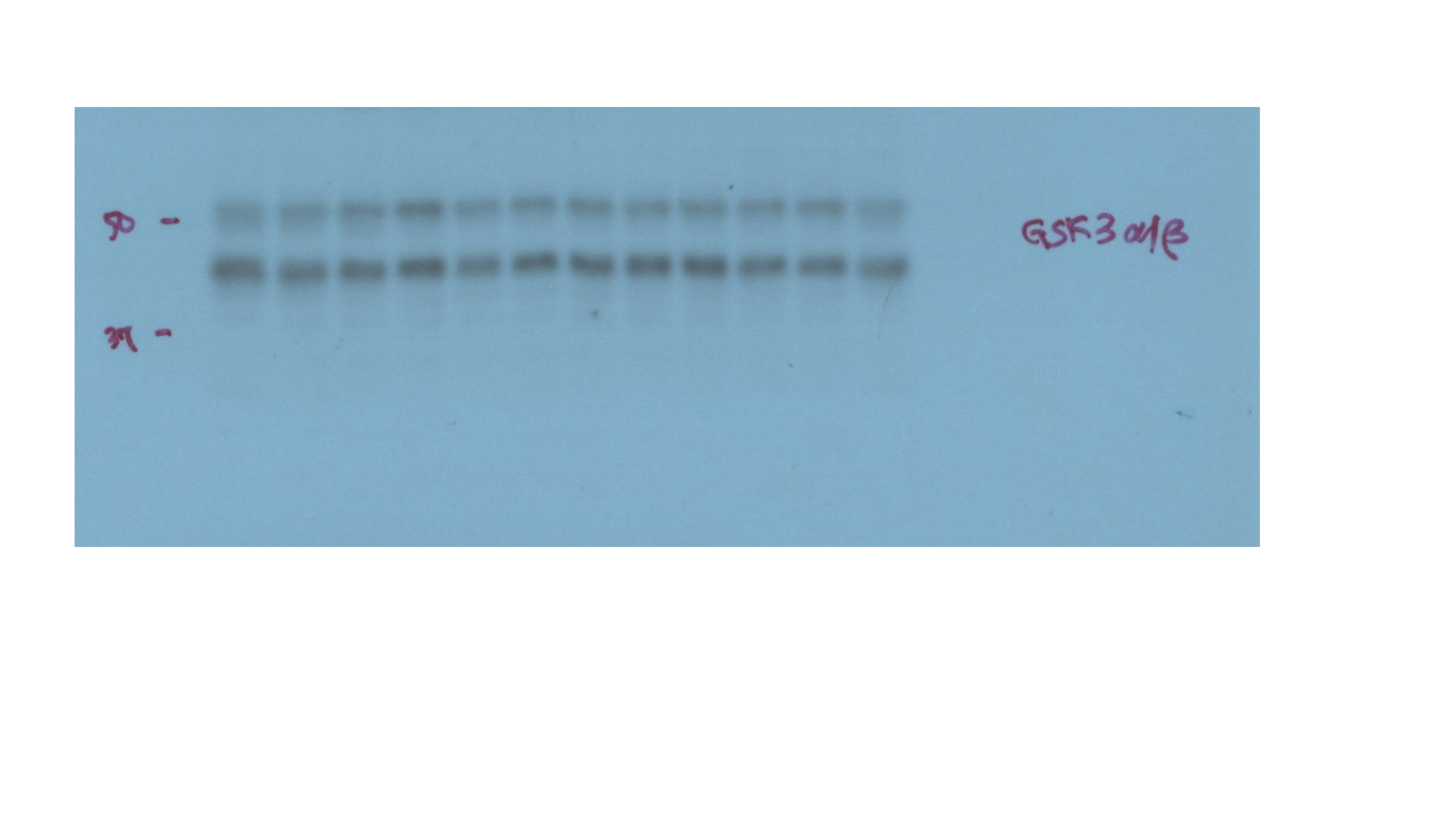

Supplement: Figure 5—source data 1. [file elife-89214-fig5-data1.zip › Figure 5 source data/9 GSK3aB raw unedited.jpg]

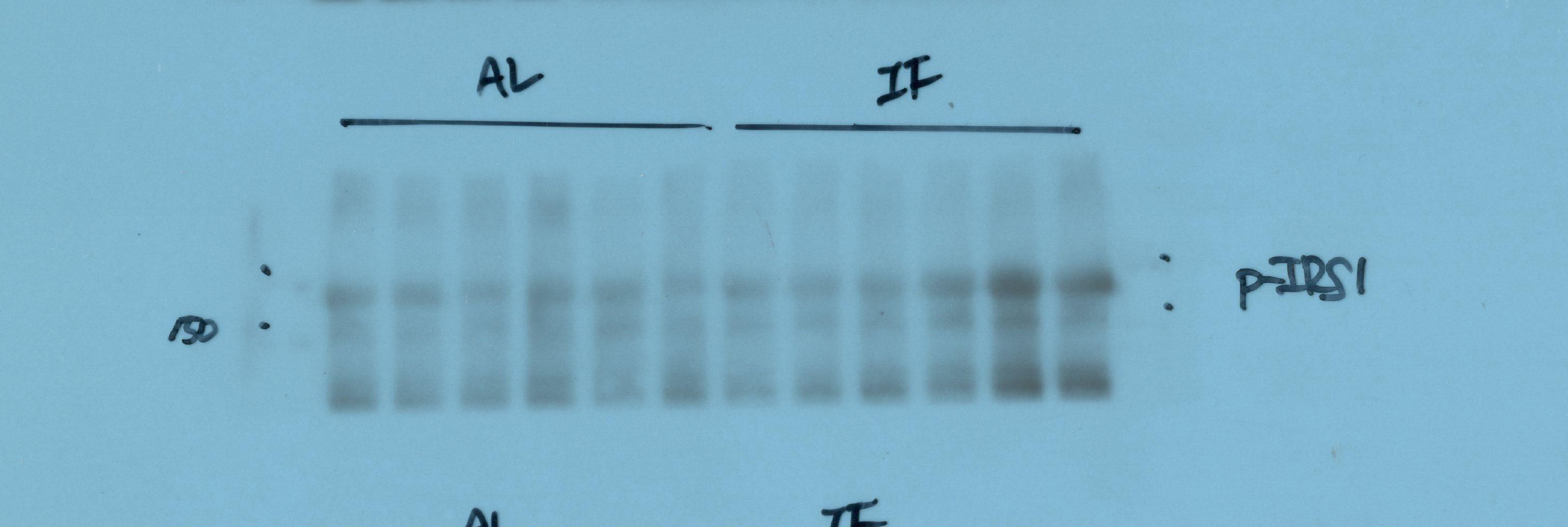

Supplement: Figure 5—source data 1. [file elife-89214-fig5-data1.zip › Figure 5 source data/12 p-IRS-1 raw unedited.jpg]

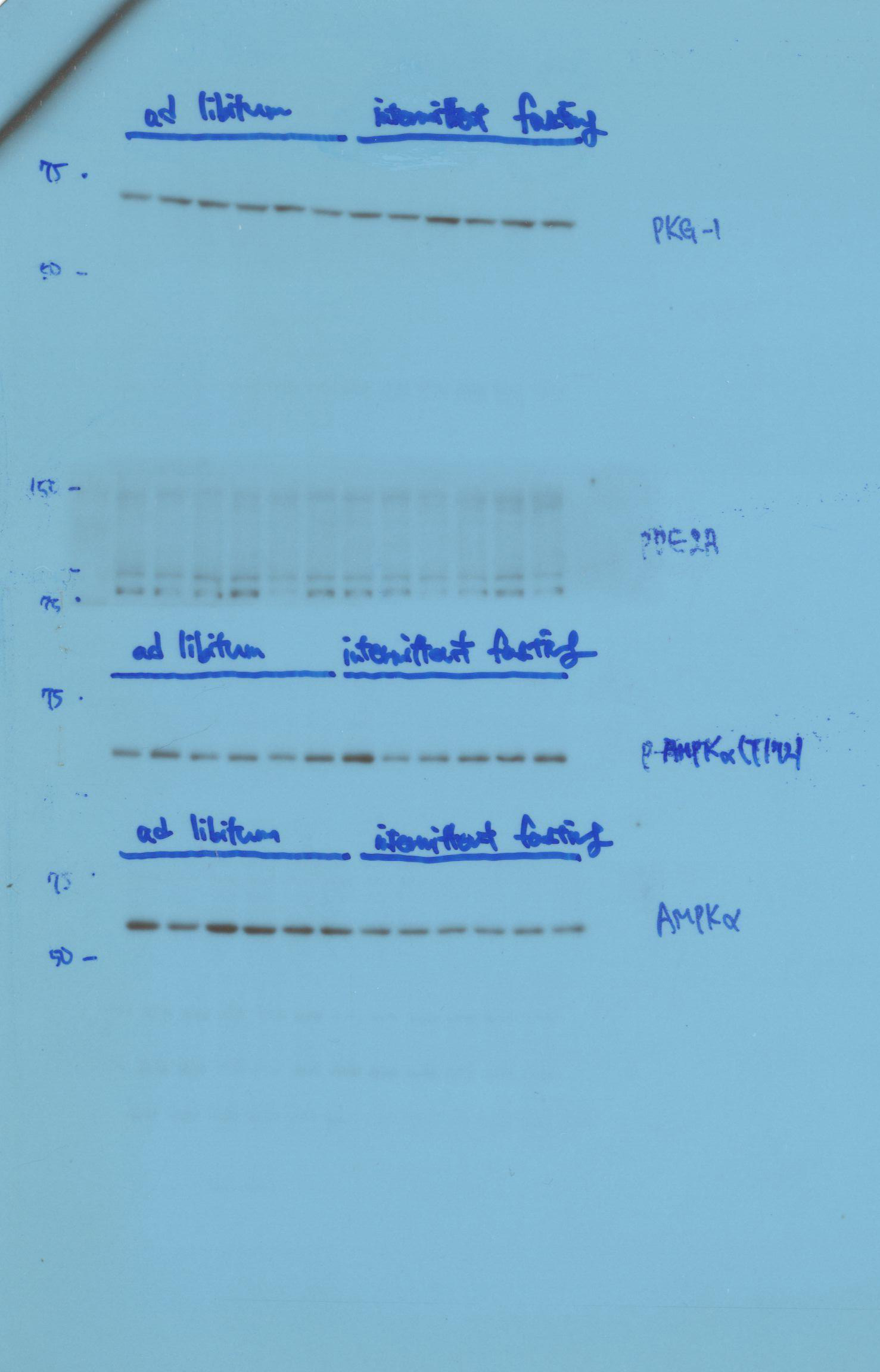

Supplement: Figure 5—source data 1. [file elife-89214-fig5-data1.zip › Figure 5 source data/1-3 p-AMPK, AMPK and PKG-1 raw unedited.jpg]

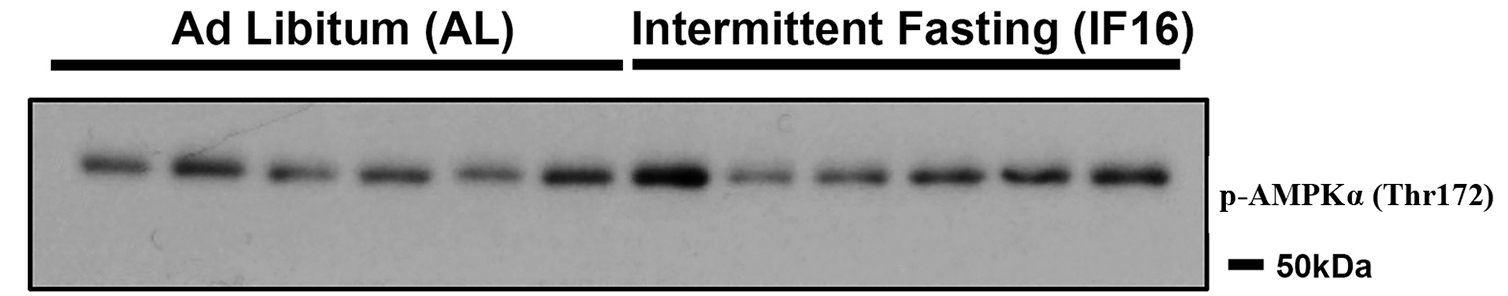

Supplement: Figure 5—source data 1. [file elife-89214-fig5-data1.zip › Figure 5 source data/1 p-AMPK.jpg]
